# Highly Porous Platinum Electrodes for Dry Ear-EEG Measurements

**DOI:** 10.3390/s20113176

**Published:** 2020-06-03

**Authors:** Max Eickenscheidt, Patrick Schäfer, Yara Baslan, Claudia Schwarz, Thomas Stieglitz

**Affiliations:** 1 Laboratory for Biomedical Microtechnology, IMTEK, University of Freiburg, 79110 Freiburg, Germany; yara.baslan@saturn.uni-freiburg.de (Y.B.); thomas.stieglitz@imtek.uni-freiburg.de (T.S.); 2 Systems Neuroscience & Neurotechnology Unit, Mindscan Lab, Saarland University of Applied Sciences, 66117 Saarbrücken, Germany; schaefer@snn-unit.de; 3 Hahn-Schickard, 79110 Freiburg, Germany; Claudia.Schwarz@Hahn-Schickard.de; 4 BrainLinks-BrainTools, University of Freiburg, 79110 Freiburg, Germany; 5 Bernstein Center Freiburg, University of Freiburg, 79104 Freiburg, Germany

**Keywords:** Ear-EEG, laser structuring, porous platinum, Berger effect

## Abstract

The interest in dry electroencephalography (EEG) electrodes has increased in recent years, especially as everyday suitability earplugs for measuring drowsiness or focus of auditory attention. However, the challenge is still the need for a good electrode material, which is reliable and can be easily processed for highly personalized applications. Laser processing, as used here, is a fast and very precise method to produce personalized electrode configurations that meet the high requirements of in-ear EEG electrodes. The arrangement of the electrodes on the flexible and compressible mats allows an exact alignment to the ear mold and contributes to high wearing comfort, as no edges or metal protrusions are present. For better transmission properties, an adapted coating process for surface enlargement of platinum electrodes is used, which can be controlled precisely. The resulting porous platinum-copper alloy is chemically very stable, shows no exposed copper residues, and enlarges the effective surface area by 40. In a proof-of-principle experiment, these porous platinum electrodes could be used to measure the Berger effect in a dry state using just one ear of a test person. Their signal-to-noise ratio and the frequency transfer function is comparable to gel-based silver/silver chloride electrodes.

## 1. Introduction

Measurement of electrography of the brain waves or muscle actions is a simple and excellent way to gain insight into a person’s condition or behavior. In particular, non-invasive methods such as electroencephalography (EEG) can provide an overview of neurobiological and cognitive states. This measurement method is relatively compact, but requires a large number of electrodes to be attached to the scalp and is usually performed in a clinical environment, as the preparation and positioning of the electrodes have a decisive influence on the usability and quality of the data. Long-term measurements over days or real-life measurements carried out by the test person are thus excluded [1]. Such a constant contact to the person would offer possibilities such as monitoring patients during sleep [2], detecting driver drowsiness [3] or attention focusing of auditory attention [4] for the improvement of hearing aids.

In order to overcome this limitation, Ear-EEG measurements have been studied in recent years, which are characterized by simple handling and unobtrusiveness [5,6,7,8]. In one approach, several electrodes are realized on an earplug and conduct the electrical cortical signal in the auditory canal and the auricle. Concepts range from flexible silicone plugs with conductive silicones or coatings [9,10,11] to hard shells that are adapted to the respective ears with individually attached electrodes that have been cut out or metal pastes that have been applied manually [2,12]. Especially, porous silver chloride is often used, which degrades over time, and conductive gel [13] must be used for acceptable measurements, which excludes long-term measurements, as this dries out over time. These gel-based silver/silver chloride electrodes are state of the art and better handling of dry electrodes does not outweigh inferior signal-to-noise levels. To enable dry electrode measurements without additional gels, the active electrode surfaces are enlarged by, e.g., iridium oxide (IrOx) and Poly(3,4-ethylenedioxythiophene)-PEDOT depositions or needle-like structures [11,14,15,16,17]. The long-term stability and the manual character of the production as well as the undefined electrode sizes and surfaces are thus the limiting factors for the accurate and reliable production and design of the ear electrodes.

In this study, different materials are compared due to their electrochemical properties and a platinum (Pt)-based ear electrode is presented with highly increased active surface with minimal impedance in the relevant frequency domain. As an interface, a noble metal is chosen such as gold, titanium or platinum, as these are corrosion resistant and many are biocompatible. Platinum is the best candidate for the application presented here [18,19], as it is not as ductile as gold [20] and does not form native oxide layers as titanium [21]. Furthermore, platinum is well known for electrodeposition processes, like nano-porous platinum [22] or platinum black [23] at a negative voltage, without strong evolution of hydrogen gas. Here, an electrodeposition process is used which creates highly porous platinum surfaces through the iterative co-deposition of Pt and copper (Cu), whereby Cu is released again after each cycle [24]. It is advantageous that the redox potential of copper is lower than the noble metal, and higher than the hydrogen potential. Thus, defined surface structures of different heights can be produced. The manufacturing process must allow a quick adaptation to the physiology of the test persons with a high degree of precision and reliability. For this purpose, prototype processes based on laser cutting were used [25], structuring individual electrode configurations for personalized earplugs. For the first tests, electrode mats with electrodes of different materials and sizes were produced. The distance between the electrodes was adapted to the personalized imprints of the ear canals and placed on earplugs. 

It can be assumed that the recording of EEG signals in and on the ear is comparable to the signals known from the scalp [26]. The measured signal amplitudes are lower, because the origin of the signals is usually not close to the ear, which requires a low noise output from the electrodes. Nevertheless, significant changes between the superior and inferior ear canal or ear canal and auricle of the signals are usually detectable. Several paradigms have already been proven [6], also in commercial products [27], especially based on frequency domain analysis. Thus, the Berger effect was chosen for validating the usability of the porous electrode material. This effect, also called the “on-off effect”, is based on very prominent potential fluctuations in a frequency range of 8−12 Hz when a person is at rest and with closed eyes. The amplitude of the waves is largest over the occipital lobe and parietal cortex, and less pronounced when approaching the frontal cortex. When the eyes are opened, the synchronization disappears, and the EEG is then dominated by small, fast beta waves with a frequency range of 14−30 Hz. It is also known to have a low likelihood of muscle contamination [28].

## 2. Materials and Methods

### 2.1. Prototype Fabrication

**Figure 1:**
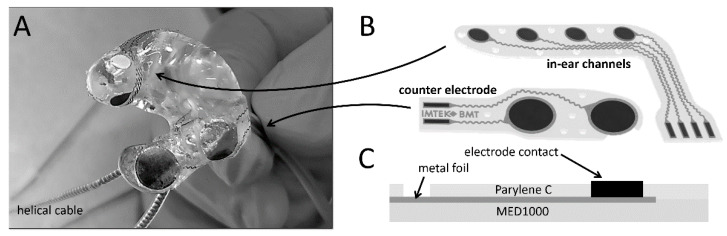
(**A**) Image of the fully assembled Ear-EEG electrode with four electrodes in the ear canal (3 mm diameter) and two large counter electrodes with a 6mm diameter. The in-ear electrode arrays are personalized laser design files with adjusted electrode spacing for an equidistant electrode spacing in the auditory channel (**B**). The two electrode mats have the same layering (**C**); a medical grade silicone laminated with a structured metal foil, encapsulated with 10 µm Parylene C.

The electrode array for the otoplastics (Figure 1A) was prepared using a fast laser-aided manufacturing process to gain highest design flexibility. The detailed step-by-step process is visualized in the supplementary Figure S1. The method used multi-layer processing to achieve the personalized design and was based on previously published processes [25,29]. It entailed the spin coating of a 70-µm-thick MED1000 silicone layer (NuSil, Carpinteria, USA) on a flat carrier substrate. Subsequently, different metal sheets (Platinum, Silver, MP35N all of 25 µm thickness) were laminated on the pre-cured silicone layer and the electrode design was cut out with a picosecond laser (Coherent Inc., Santa Clara, CA, USA), using the parameters suited for the material type and thickness. The excess metal was peeled off and the silicone layer fully cured, forming a strong bond with the metal structures. The conductor paths were designed in a meandering shape in order to maintain the high flexibility of the system (Figure 1B). A topside coating was performed with a chemical vapor deposition system (Dimer DPX-C, Coater PDS 2010, Speciality Coating Systems Inc., Indianapolis, IN, USA) with 10 µm Parylene-C (Figure 1C). The prototyping laser process was used again to pattern the Parylene-C layer to expose the active sites of the electrodes on one end, and the connector soldering pads on the other, while leaving the conducting paths between them isolated. Finally, the outlines were cut with the same laser, to release the probe. 

### 2.2. Soldering, Cleaning and Characterization of Electrodes

After fabrication, the electrode arrays were assembled with a connector by soldering enameled copper or MP35N helical winded cables to the soldering pads, and the single wires to a pin header for addressing each electrode separately. The electrode array was cleaned thoroughly with ethanol and de-ionized water to remove residues or dust particles from the surface. In addition to washing, the electrodes were also cleaned electrochemically by running 15 cycles of cyclic voltammetry at a rate of 250 mVs-1 within a range of −600 mV to +900 mV in a phosphate-buffered saline solution. All electrochemical procedures were performed with a three-electrode setup using a potentiostat and a frequency analyzer (Solartron 1260&1287, Solartron Analytical, Farnborough, UK). The assembled electrodes were characterized before and after a subsequent electrodeposition, in order to estimate their effective surface area as well as to analyze their frequency response. To estimate the area, cyclic voltammetry was performed for three cycles at a rate of 100 mVs^−1^ within a window of −700 to +1000 mV vs. SCE. The evaluation of the charge delivery capacity (CDC) was performed with Origin (OriginPro 2019, Northhampton, MA, USA) by calculating the enclosed polygon area of the last cycle. As for the frequency response, the impedance in a range of 0.1 Hz to 100 kHz was determined, at a rate of five steps/decade and a 20-mV AC amplitude. Scanning electron micrographs of the electrode morphology and focused ion beam cuts were made with a Scios 2 (Thermo Fischer Inc., Waltham, MA, USA). The chemical analysis of the surfaces was performed by an X-Ray Fluorescence Spectrometer (µ-XRF M4 Tornado, Bruker Corporation, MA, USA) in a vacuum. The beam had a strength of 50 kV at 600 µA and a spot size of 20 µm. After 30 s measurement, the atomic concentrations were analyzed with the internal software.

The finalized electrodes were glued onto personalized otoplastics. Ear impressions of the respective test person were used to produce hard shells from acrylic resin (Sivantos GmbH, Erlangen, Germany). The shells were cleaned with isopropanol and the silicone side of the electrode mat was bonded to the shell with ethyl-2-cyanoacrylate adhesive (RS Components SAS, Corby, UK). Two large electrodes were positioned in the concha and four smaller ones evenly distributed around the ear channel section (the electrode design were adapted to the otoplastic). The electrodes were placed on the outer edge of the earpiece in order to allow an electrode measurement as deep as possible in the ear channel. The connections of the mats and the cables were fixed on the outside of the shell with MED1000.

### 2.3. Electrodeposition and Electroplating

The electrodeposition process used in this work was adapted from Frei et al. [24], with minor changes to suit the application. The experiments were conducted in a three-electrode setup using the Solartron Analytical 1287A potentiostat function. The reference electrode was a saturated calomel electrode (SCE, KE 11, Sensortechnik Meinsberg, Ziegra-Knobelsdorf, Germany) and for the counter electrode a platinum mesh (0.06 mm wire diameter/99.9%, Chempur, Karlsruhe, Germany) connected to a platinum wire (0.1 mm diameter, Chempur, Karlsruhe, Germany) was used. The electrolyte consists of 0.5 M sulfuric acid (TitriPUR, Merck, Darmstadt, Germany) containing 0.02 M Cu_2_SO_4_ (Merck, Darmstadt, Germany) and 0.02 M H_2_PtCl_6_ (Chempur, Karlsruhe, Germany). During the experiment, the electrolyte was kept in a nitrogen atmosphere, which was established by first bubbling nitrogen through the solution for 20 min and then purging it continuously on the surface of the electrolyte. Once the solution was saturated with the nitrogen, the electrodeposition process was executed by simultaneously depositing Cu and Pt from the electrolyte, by applying a deposition potential of −500 mV vs. SCE for 9 s to the working electrode. The deposition was followed by a selective dissolution of copper at a potential of +700 mV vs. SCE for 4.5 s. This alternating process was repeated 50 times. To counteract possible accumulation of gas bubbles on the active surface, the medium was constantly moved with a magnetic stir bars.

Silver electrodes were chlorinated in a potassium chloride solution (3 moL) with a single Ag/AgCl counter electrode, by passing a DC-current through the electrode at a rate of 1 mA/cm^2^ of surface area for 15 s.

### 2.4. In-Ear Electroencephalography Measurement

Before each experiment, the auricle and the anterior part of the external auditory canal of the test person were cleaned with a cotton swab and alcohol. This area was then treated with a peeling gel using a cotton swab to remove old and dead skin residues. Finally, the exfoliating gel was thoroughly removed and the area was cleaned again with alcohol. In the case of Ag/AgCl probes, gel was applied to the electrode surfaces in a dot-shaped manner. The prepared electrode was then inserted into the prepared ear and the electrodes connected to the EEG amplifier (g.USBamp, g.tec medical engineering GmbH, Austria). During the measurement, the subject sat quietly on a chair in a quiet and non-illuminated room. In a first measurement, the test person opened his eyes for more than 30 s and fixes a cross on the wall in front of him. In a second measurement, the respondent closed his eyes for 30 s. At the same time, the EEG was recorded at a sampling rate of 512 Hz. The spectral density was analyzed by the multitaper method [30]. The time signals of all single channels were filtered with a set of Slepian sequences of a window half bandwidth of 4 and the final spectrum was obtained by averaging all convoluted data. The band powers were calculated by integrating a parabolic approximation between the sample points of a chosen frequency spectrum (Simpson′s rule). All calculations were performed with customized programs in Python. 

## 3. Results

### 3.1. Electrode Preperation

A laser-assisted ablation process realizes the patient-specific manufacture of the electrode arrangement. For an equidistant arrangement of the four electrodes in the ear canal, the radius of the personalized otoplastic was first measured and the electrodes structured accordingly on the silicone mat. The measuring electrodes with a diameter of 3 mm were intended to be placed in the external auditory channel and two further counter electrodes with a diameter of 6 mm in the concha at the level of the cymba. The electrode placement was based on known experimental measurements and simulations, which predict the greatest potential drop at the ear [26]. The conductive paths were guided in a meandering pattern to minimize stress on the conductive paths when they were attached to the hard shell. The solder joints between the electrode mat and the helically wound cable were fixed and insulated with epoxy adhesive (Figure 1). The highly flexible cable relieved the tension on the fixation and was not as susceptible to movements as straight cables.

**Figure 2:**
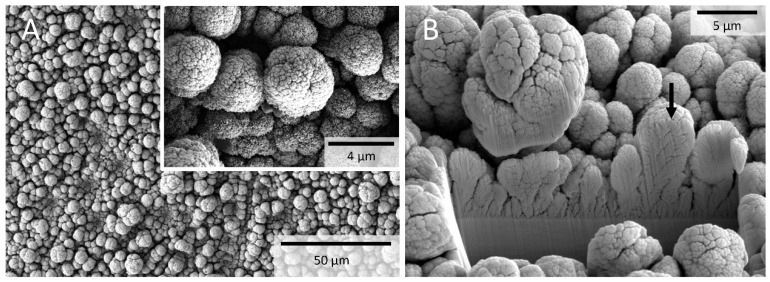
Scanning electron microscopy of the full-surface metal coating with porous platinum after alloying with copper and alternating dealloying. The surface shows different forms of growth (**A**). In deeper layers, finger-like structures can be seen, which become more cauliflower-like towards the top (insert). A cross-section with the focused ion beam shows channel structures up to the platinum foil (**B**). The height of the average structures (arrow in (**B**)) is around 10 µm.

Before the polymer-based electrodes were glued onto the earplug, the surfaces were treated and, in particular, the platinum-copper alloy was deposited. This results in different microstructures that were created depending on the number of cycles of iterative alloying and delegation of platinum and copper. A deposition with 50 iterations has proven to be advantageous to obtain a 5- to 20-µm-thick layer of platinum. The structures change from initially a bicontinuous ligament-channel structure to larger cauliflower-like structures (Figure 2).

XRF studies show a high copper content of 12 wt% in the applied layer despite alternating dealloying (see Table 1). This layer cannot be spatially assigned to any structure and was also not optically recognizable. Two attempts to reduce the copper content were made without any effect. On the one hand, the negative potential of −600 mV vs. SHE was kept for 30 min after deposition; on the other hand, the electrodes were etched in a standard etching bath for printed circuit boards with heated (80 °C) sodium persulfate (Na_2_S_2_O_8_) for 20 min. Subsequent XRF measurements show only small variations that were not significant.

### 3.2. Electrochemical Electrode Characterisation

**Figure 3:**
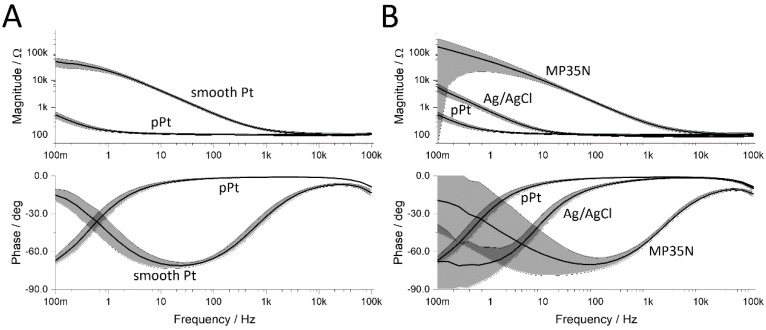
Electrochemical impedance measurement of different electrode materials. (**A**) Smooth platinum electrodes before and after electrochemical deposition of porous platinum (N = 10). (**B**) comparison of MP35N (N = 7), Ag/AgCl (N = 7) and porous platinum (pPt) electrodes of the same geometric area (3 mm diameter). Cutoff frequency of the porous platinum electrodes are orders of magnitude lower than those of smooth platinum (**A**) and show even lower cut-off frequencies than state-of-the-art silver/silver-chloride electrodes (**B**) or common metal alloys (MP35N).

All electrodes were built in the layered structure of PDMS—metal foil—Parylene C (Figure 1C) in different designs, whereby the accuracy of the ablative laser process gave the real spot sizes. The properties of the electrodes were determined in PBS solution to simulate a physiological measurement condition (Figure 3). Smooth platinum electrodes show a typical impedance behavior of a Randles cell with the access resistance at high frequencies and a characteristic increase in impedance to low frequencies given by the active surface and electrode matter (Figure 3A). The access resistance is given by the geometric expansion of the electrode and therefore it is independent of the electrode material or the roughening (Figure 3A,B). The deposition of platinum copper or the chlorination of the electrodes was performed using the Solartron setup. The porous surfaces show a significant reduction of the impedance at low frequencies, which leads to a purely resistive behavior at frequencies higher than 1 Hz (Figure 3A, phase). A similar behavior can be observed in the chlorination of silver foils, whereas the reduction of impedance was not as strong as in the surface enlargement by platinum. The reference material MP35N, which is the gold-standard material due to its biocompatibility and stability, has an even earlier increase in complex impedance than smooth platinum foils.

**Figure 4:**
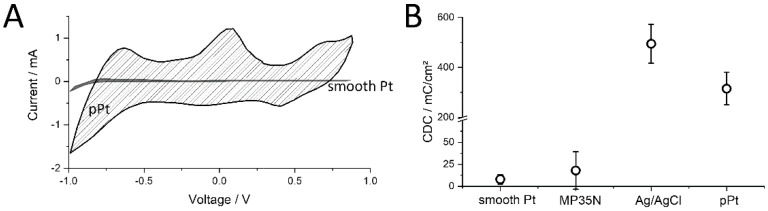
Cyclic voltammetry measurement. (**A**) Smooth platinum electrodes before and after electrochemical deposition of porous platinum (mean of 10 electrodes each). (**B**) Comparison of the charge delivery capacity (CDC) of MP35N (N = 7), Ag/AgCl (N = 7), smooth and porous platinum (pPt) electrodes (N = 10 each) of the same geometric area. Mean and standard deviation.

Another electrochemical characterization of the electrodes is cyclic voltammetry, which indicates the current flow at a linearly varying voltage between two reversal points (Figure 4). Here, the dissipative and reactive currents were superimposed across the electrode interface. Both components depend on the effective surface area of the electrodes and increase with the roughness of the surface (Figure 4A). A quantitative evaluation of electrochemical electrode surface properties is the charge delivery capacity (CDC), which is calculated by the enclosed area of the curve of a cycle and is divided by the slope of the applied ramp to obtain a value for the charge transfer in the applied voltage window. These values were normalized to the geometric electrode size. A comparison of CDCs shows large differences between the materials used and surface conditions (Figure 4B). For example, the rolled and smooth surfaces of platinum and MP35N show a CDC of 8 and 18 mC/cm², respectively. A surface enlargement of the porous platinum electrodes results in a 40-fold enlargement of the CDC to 315 mC/cm². Silver/silver chloride electrodes also showed a strongly enlarged CDC (495 mC/cm²) too, which was due to the change in surface structure, but mainly to the reactive property of the metal salt in contact with the electrolyte.

### 3.3. Ear-EEG Measurment

**Figure 5:**
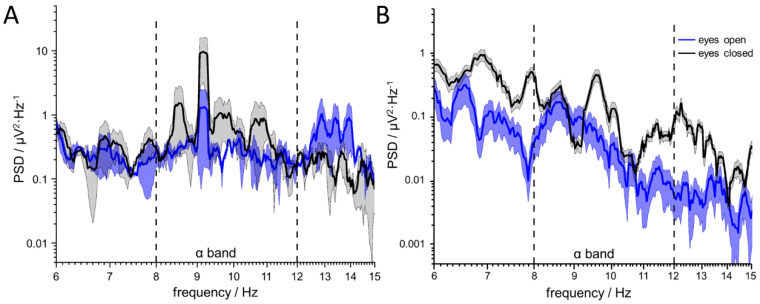
Averaged frequency spectra of four electrodes in the auditory canal and their standard deviation (transparent interval). The power density with closed eyes (black) is higher in the alpha band between 8 and 12 Hz than with open eyes (blue curve). This Berger effect can be observed with chlorinated silver electrodes using conductive gel (**A**) that rely on state of the art technology in EEG measurements in general as well as in the new approach and dry porous platinum electrodes (**B**) that has been developed in this work.

A promising application for the greatly enlarged electrode surfaces is the recording of biological signals in contact with the skin. As proof of principle, the EEG signals were recorded at the auricle and in the ear canal. Two pairs of ear EEG electrodes were made for one person, one with platinum-copper alloy and one made of chlorinated silver foil. The ear molds were easy to use and can be inserted by the individual independently. Towards a real-world scenario of the platinum electrodes, external reference and counter electrodes, e.g., on the lobulus, Cz, or low mastoids have been omitted. The data refer exclusively to measurements from four electrodes within the auditory canal referenced to the electrodes in the concha on the same side of the head. To check the functionality, the Berger effect was measured in a shielded EEG laboratory without external disturbances, where the test person kept the eyes open and closed for 30 s each. In both conditions the power of the alpha-waves—a frequency band between 8−12 Hz changes [31]. At rest and with closed eyes, these waves can be derived from the entire scalp, originated in a synchronization of both brain hemispheres. When the eyes are opened, the synchronization disappears and a blockage of the alpha-rhythm occurs. A successful differentiation of the frequency spectra in the alpha band [8−12 Hz] was possible with both materials, whereas conductive gel had to be used with silver/silver-chloride electrodes while measurements with the porous platinum electrodes were conducted in a dry condition. Furthermore, for the measurements with the silver electrodes, the low mastoid on the same side of the head was used as a reference. The mean values and standard deviations of the frequency spectra across all four electrodes in the auditory canal show an increase in power in the alpha band when the eyes were closed (Figure 5). Thus, on average, an increase in power of 2.4 ± 1.8 µV² can be expected for chlorinated silver electrodes and 4.5 ± 3.7 µV² for porous platinum electrodes. The high variances reflect the different electrode performances. For example, a selected electrode with a ratio of up to 4.4 can be found in the chloride silver device, whereas the electrodes in the platinum system had a median change of 2.43 power in the alpha band (Table 2). In general, however, the power density of silver electrodes was one order of magnitude higher than that of porous platinum electrodes. Additionally, a change of the increased power in the beta band with open eyes was only visible with the silver electrodes. On the other hand, the signal variance of the platinum electrodes was significantly lower.

## 4. Discussions

The two-step process presented here for manufacturing earplugs with passive electrodes for EEG measurements was designed for rapid personalization. Depending on the hard shell of the test person, the electrode spacing and positioning can be freely selected and can be produced in the prototype laser process with little effort. The computer-aided manufacturing thus offers more design freedom and better control than manual printing processes and cabling [6,9]. The small thickness of the entire electrode mat allows the otoplastic to be worn comfortably because it does not protrude as far as printed electrodes do. The 10-µm-thick cover layer also allows good contact with the skin-especially in the case of rough electrodeposited platinum whereas the insulation between the individual electrodes was maintained. The large thicknesses of the layers are a great advantage compared to alternative coatings such as iridium oxide (IrOx) [32], as a flat surface of electrode material and insulator can be achieved. In a second step, the various post-treatments of the electrodes, the connections and the positioning on the earplug are independent of production, which also allows a higher degree of freedom from the choice of material to the overall concept. The combination of the electrodes with the highly personalized otoplatics is beneficial, since the dry electrodes are sensitive to movement and thus to changes in contact and position [33]. This motion tolerance of the system is a key feature for a commercial application and the success of daily-life applications [27]. This development offers new application scenarios but does not replace well-established standard EEG measurements on the scalp.

The surface enlargement by the iterative electroplating of platinum and copper and the subsequent dealloying by removal of copper atoms leads to a strong enlargement of the active surface of the electrodes. The area of the cyclic voltammetry measurement shows a magnification by a mean factor of 39.41. In previous studies, a higher value of up to a factor of 1000 was shown, whereby these values were determined by hydrogen or carbon dioxide stripping [24] which is a factor 5 higher than other electrodeposition techniques [22]. The measurement presented here was intentionally performed with PBS and a higher scan rate of 100 mV/s as this provides a better insight into the effective surface in the application scenario. In the intended EEG measurement system, dissolved salts in the form of sweat also form the decisive phase boundary. Due to their larger shape and hydrate shell, however, salts have a much larger dimension than hydrogen ions and therefore only a fraction of the porous surface contributes to the interface. The Faraday resistance of the electrode-which is, e.g., a chemical reaction of platinum with hydrogen or oxygen-was negligible in the relevant frequency range (compare impedance measurements). The capacitance of the electrode has been increased so that it short-circuits the phase boundary and only the access resistance of the electrolytes was of importance. The access resistance depends on the geometric shape and the composition of the electrolyte, namely the salt concentrations. The noise of the electrode is given by the real part of the complex electrochemical impedance, which here has been reduced to the theoretical minimum starting from 1 Hz. Therefore, this capacitive interface is as good as any other material (e.g., silver chloride) even at low frequencies. The transition to the skin and the associated additional (noisy) resistance is thus the limiting factor [34]. After an initial drift of the electrodes, however, a stable measurement with a performance comparable to that of gel should be possible [33]. The naturally high offset caused by the electrochemical potential of platinum must be taken into account in the measurement amplifier [20]. Thus, these dry electrodes might become an alternative to wet ones but their performance has to be evaluated on the single application case. Transfer of performance statements between different application scenarios, e.g., in-ear vs. scalp EEG, is not permitted. Careful characterization and evaluation have to be done for each application separately.

All plated electrodes (diameter 3 mm) used here were below 5 kOhm at 20 Hz and therefore compatible with standard medical EEG systems. In this study, platinum was compared to MP35N nickel-cobalt base alloy, which is widely used in biomedical applications, which has an even higher impedance in the rolled state than platinum. Silver/silver-chloride electrodes, which are used as conventional EEG electrodes, show comparable electrical characteristics to the porous platinum. However, silver and gold electrodes should only be used about 10−20 times [35]. The silver electrodes were not homogeneously electroplated and therefore only a slight improvement of the EIS can be seen. Even if the charge capacity were the highest, what would be expect for a non-polarizable electrode. The increase in impedance at low frequency was therefore partly due to the material [35] and partly to the inhomogeneous coating. Furthermore, silver/silver chloride was heavily affected by exposing it to sunlight. Which requires special storage of the electrodes. A promising surface enlargement of the electrodes with iridium oxide for an application as dry electrode was also used, whereby only a surface enlargement by a factor of 15 is to be expected here [32]. These nano-porous, very thin layers (as well as platinum [22,32] or carbon nano tubes [7,9,10]) show excellent properties as implantable electrodes, especially having small diameters. However, they may not have the necessary stability and thickness to provide a good and long-lasting contact as a re-usable patch electrode, which is to be expected with the microstructure of the porous platinum proposed here. Even though platinum films, unlike gold, have excellent mechanical stability [20], further tests should investigate the influence of friction, electrical changes in long-term applications [36] and initial drifts due to sweating [33].

The XRF measurements show a significant copper content in the bulk of the electroplated material, which remains in the metallic compound. Excessive and selective etching of copper (chemical and electrochemical) shows no change, so it is suspected that the copper atoms were not exposed on the surface of the porous material but were firmly enclosed by metal lattices. For long-term applications of the material in contact with the skin, a low concentration of copper is nevertheless to be assumed, which could get into the body percutaneously [37] and lead to irritation or allergies. However, pure copper material is commonly used as intrauterine device to regulate fertility or as dental implants, and if the concentrations are very low as in the current study the risk to the patient is minimal. With an overestimation volume of the grown platinum copper alloy of 0.28 mm³ (10-µm-thick structure) a recording spot has a maximum of 0.78 mg copper. For comparison, the Food and Nutrition Board gives a NOAEL value of 10 mg/d for an adult. In a possible application, however, further tests must be carried out.

A further possible application for the flexible thin electrode mats are patch electrodes for the skin. Measuring the in-ear EEG was chosen, as this is a highly interesting location, which on the one hand is easily accessible hub of information and on the other hand makes high demands on the personalization of the device [38]. The measurements of the Berger effect were successful, although there were some differences to the known signals [39], which are normally picked up over the entire head area (C3 or C4 and adjacent electrodes). The relative increase of power of alpha waves during eyes closed in comparison to eyes open was significantly greater than one with both materials. With an acceptable median value of 2.5, the Berger effect could be demonstrated with the dry electrodes, which measured exclusively in ear. The ratio is significantly lower than standard EEG electrodes, which make recordings over the entire head, like gold-coated commercial dry electrodes (g.Sahara) with a measured ratio of 10 at electrodes O1 and Oz [39]. However, comparable products on the ear have a lower ratio of 2.5 dB, which is a factor of 1.3 [5]. The choice of the reference electrode on the same ear in the auricle is an additional challenging choice [40]. The derivation of the EEG signal referring to, e.g., the mastoid, gives better results like in the case of the chlorinated silver electrodes. However, the silver electrodes showed unsatisfactory measurement results in dry condition. Further improvements can be expected if the electrodes of both ears are used and the signals are referenced to the other side of the head [40]. 

The roughened planar electrodes are therefore ideal for measurements on the skin when there is less hair to prevent close contact with the skin [34]. Thus, a variety of other measurement of electrical biosignals are possible, such as electrocardiogram (ECG), electromyogram (EMG), and electrooculogram (EOG). Since the approximate toxicity of the electrodes is also low, the electrodes can also be used as transient micro-electrocorticography in direct contact with brain tissue [41].

## 5. Conclusions

Towards a reliable and resistant electrode material for skin patch electrodes with a high signal to noise ratio, the platinum-copper alloy presented here is a promising candidate. The simple and controllable production of the thick coatings by electrochemical processes on personalized structured metal foils is a decisive advantage over costly cleanroom processes and deposition of mostly thin functional layers. As dry EEG electrodes, the porous platinum layers show excellent measuring properties, especially in the relevant frequency range from 0.1 to 500 Hz. Beyond 1 Hz, the theoretical minimal impedance for electrodes with a diameter of 3 mm was achieved, thereby also obtaining the lowest possible noise output. The interfacing properties for in-ear EEG applications were further improved by applying the electrodes to personalized otoplatics for close skin contact and resistance to movement. Alternative materials such as smooth platinum, MP35N or chlorinated silver foils have higher impedances and coupling properties in the relevant frequency band. The chemical inertness and the low degradation of the material compared to, e.g., chlorinated silver electrodes, suggests a long-lasting and reliable use of the material.

## Figures and Tables

**Table 1:** XRF Measurement of three pristine platinum electrodes with electrodeposited platinum-copper alloy. Two of these electrodes where treated with different etching methods for copper (electrochemical dealloying and wet chemical etching).

Electrode Treatment	Pt [wt%]	Cu [wt%]
pristine	87.16 ± 0.09	12.84 ± 0.09
Dealloying 30 min	88.60	11.40
Na_2_S_2_O_8_ 20 min	87.83	12.17

**Table 2:** The alpha power is calculated in the bandwidth between 8 and 12 Hz for 30 s closed eyes. The Berger effect is the ratio between the alpha powers of closed and opened eyes.

Porous Pt	α Power [µV²]	Berger Effect
channel 1	0.603	2.43
channel 2	0.643	2.28
channel 3	0.606	2.44
channel 4	0.362	10.9

## Supplementary Materials

The following are available online at https://www.mdpi.com/1424-8220/20/11/3176/s1, Figure S1: Step-by-step manufacturing process.
