# Supplementary material for: Highly Porous Platinum Electrodes for Dry Ear-EEG Measurements
Source: Sensors (Basel). 2020 Jun 3;20(11):3176. doi: 10.3390/s20113176 (PMC7309044; doi:10.3390/s20113176)
Supplement: Supplementary file 1 [file sensors-20-03176-s001.pdf]

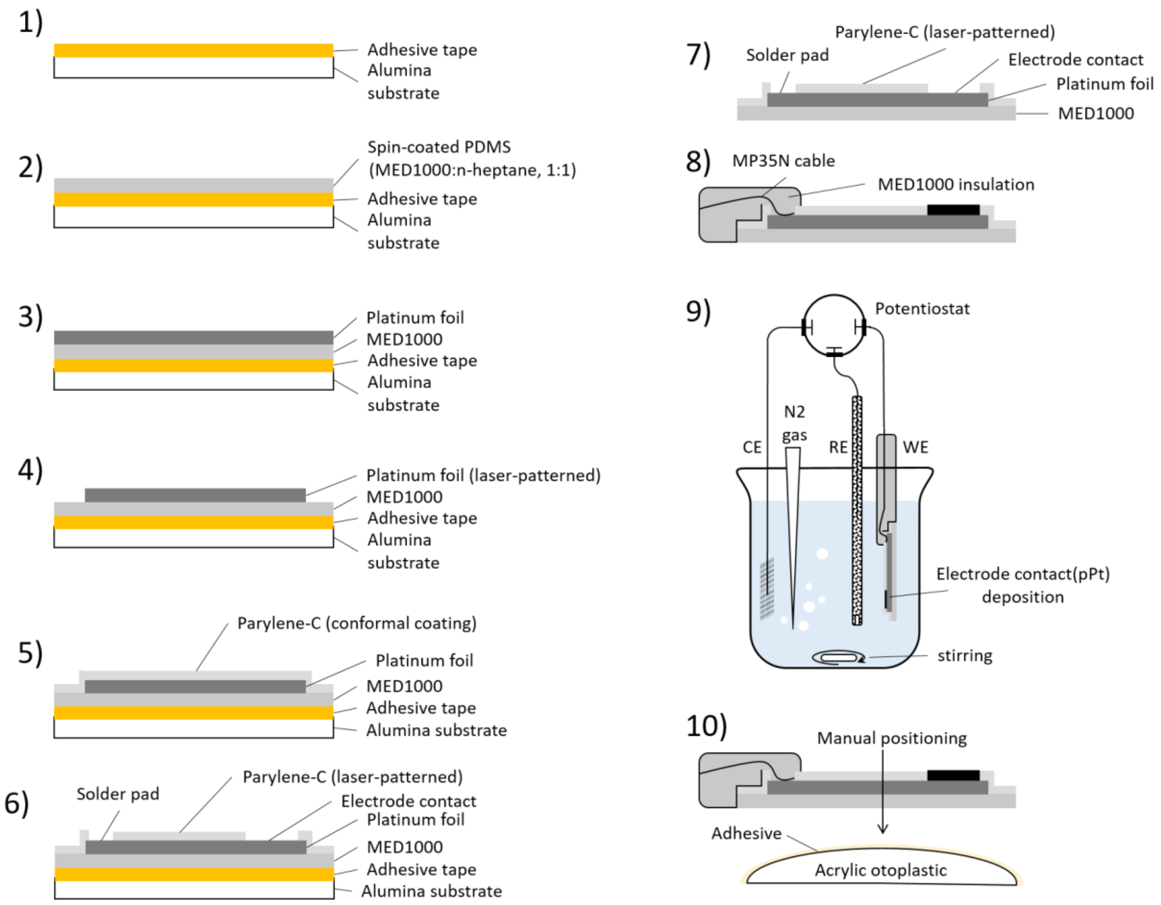

Figure S1: Step-by-step manufacturing process of personalized EEG electrodes. On a flat aluminium substrate with Tesa-tape (1), the first layer of MED1000 is spin-coated (2) and in a semi cross-linked state a 25µm thick platinum foil is rolled on top (3). The metal surface is structured and the unwanted metal is removed. Afterwards, the entire structure is encapsulated with Parylene C (5), in order to laser openings into it (6) and define the outer perimeters. The sample can be removed from the carrier substrate (7) and a cable is assembled and encapsulated with MED1000 (8). Between the individual production steps, which are all carried out in a clean room environment, the surfaces are repeatedly cleaned with isopropanol to remove residues from the laser process. (9) The electrode was then cleaned (cyclic voltammetry) and coated in a potentiostat in a three electrode configuration. A saturated calomel electrode served as the reference electrode and a platinum mesh served as the counter electrode. The deposition electrolyte consists of sulfuric acid, Cu2SO4 and H2PtCl6. During the experiment the electrolyte was kept at a saturated nitrogen atmosphere. To avoid the deposition of hydrogen bubbles on the electrode surface, the electrolyte was constantly moved with a stir bar. After the electrode was characterized in PBS and rinsed with DI water and isopropanol, it could be glued to the cleaned otoplastics (10).
